# Click-chemistry approach to study mycoloylated proteins: Evidence for PorB and PorC porins mycoloylation in *Corynebacterium glutamicum*

**DOI:** 10.1371/journal.pone.0171955

**Published:** 2017-02-15

**Authors:** Hanane Issa, Emilie Huc-Claustre, Thamila Reddad, Nolwenn Bonadé Bottino, Maryelle Tropis, Christine Houssin, Mamadou Daffé, Nicolas Bayan, Nathalie Dautin

**Affiliations:** 1 Institute for Integrative Biology of the Cell (I2BC), CEA, CNRS, Univ. Paris‐Sud, Université Paris‐Saclay, Gif‐sur‐Yvette Cedex, France; 2 Holy Spirit University of Kaslik (USEK), Jounieh, Mount Lebanon, Lebanon; 3 Institute of Pharmacology and Structural Biology (IPBS), UMR 5089, France; Centre National de la Recherche Scientifique, Aix-Marseille Université, FRANCE

## Abstract

Protein mycoloylation is a recently identified, new form of protein acylation. This post-translational modification consists in the covalent attachment of mycolic acids residues to serine. Mycolic acids are long chain, α-branched, β-hydroxylated fatty acids that are exclusively found in the cell envelope of *Corynebacteriales*, a bacterial order that includes important genera such as *Mycobacterium*, *Nocardia* or *Corynebacterium*. So far, only 3 mycoloylated proteins have been identified: PorA, PorH and ProtX from *C*. *glutamicum*. Whereas the identity and function of ProtX is unknown, PorH and PorA associate to form a membrane channel, the activity of which is dependent upon PorA mycoloylation. However, the exact role of mycoloylation and the generality of this phenomenon are still unknown. In particular, the identity of other mycoloylated proteins, if any, needs to be determined together with establishing whether such modification occurs in *Corynebacteriales* genera other than *Corynebacterium*. Here, we tested whether a metabolic labeling and click-chemistry approach could be used to detect mycoloylated proteins. Using a fatty acid alkyne analogue, we could indeed label PorA, PorH and ProtX and determine ProtX mycoloylation site. Importantly, we also show that two other porins from *C*. *glutamicum*, PorB and PorC are mycoloylated.

## Introduction

Fatty acid acylation of proteins is a post-translational modification found in all domains of life [1]. In eukaryotes, proteins can be *N*-myristoylated, *S-* or *N*-palmitoylated, palmitoleoylated (modified on serine or threonine residues), *S*-prenylated, cholesteroylated and/or glycosylphosphatidylinisotol (GPI)-linked. In these organisms, lipidation facilitates the association of proteins with membranes and other proteins, mediates protein trafficking and regulates protein stability and activity [2]. In prokaryotes, the term “lipoproteins” describes a large family of functionally diverse exported proteins, which are lipidated on a conserved N-terminal cysteine residue. The biogenesis of these proteins has been extensively studied and the proposed role for lipoprotein acylation is to anchor proteins to bacterial membranes [3]. In addition to lipoproteins, other “non-canonical” acylated proteins have been reported in prokaryotes. These include repeat-in-toxin exoproteins from Gram-negative bacteria, which are acylated on the ε-amino group of internal lysine residues [4] and one example of internally *S-*palmitoylated protein from *Erwinia carotovora* [5]. These "non-canonical" lipoproteins are secreted virulence factors playing important roles in the pathogenicity of the producing bacteria [4,5].

Recently, a completely new form of protein lipidation has been discovered in bacteria. Specifically, 3 proteins from *Corynebacterium glutamicum* (PorA, PorH, ProtX) were found to be covalently modified with mycolic acids (MA) and PorA orthologues were similarly modified in *C*. *efficiens* and *C*. *diphtheriae* [6]. This post-translational modification occurs on serine residues [6,7] and hence represents the first example of protein *O*-acylation in prokaryotes. It is also the first occurrence of protein modification by mycolic acids (“mycoloylation”). Mycolic acids are long chain (C30-C100), α-branched, β-hydroxylated fatty acids exclusively found in the envelope of *Corynebacteriales*. This order, which includes genera such as *Corynebacterium*, *Mycobacterium*, *Rhodococcus* or *Nocardia*, is indeed characterized by an atypical envelope where the plasma membrane is surrounded by a thick layer composed of a covalent tripartite macromolecule core, namely the peptidoglycan-arabinogalactan-mycolic acids complex, itself interacting with non-covalently attached lipids, which include mycolic acids esterifying trehalose [8,9]. The mycolic acid-containing part of this complex is functionally similar to the outer membrane of Gram-negative bacteria and is usually referred to as the “mycomembrane”. The mycomembrane is highly impermeable to antimicrobial compounds but its organization and protein composition is still poorly defined [10]. Among the few proteins identified so far as mycomembrane proteins, most display porin activity *in vitro* [11]. The *M*. *smegmatis* Msp porins, which are necessary for optimal growth of the bacteria, fold as octameric goblet-like pores made of two consecutive 16 β-stranded barrels, with a central 10 to 48Å-wide channel [12,13]. *M*. *tuberculosis*, which does not possess Msp homologues, expresses a protein (OmpATb (Rv0899)) that shows sequence similarity with the Gram-negative bacteria OmpA porin. OmpATb is required for virulence but the actual function of OmpATb as a porin *in vivo* is still unresolved [14,15,16]. The OmpATb N-terminal domain, found as sufficient for *in vitro* pore-forming activity, folds as an α+β sandwich, with 3 α-helices packed on the same side of a flat β-sheet formed by 6 parallel/antiparallel β-strands [17,18]. According to this structure, the protein would not be able to form a membrane channel unless oligomerization occurs [17]. Finally, another surface-accessible protein (Rv1698), conserved in all mycolic-acid containing bacteria, displays channel-forming activity *in vitro* and is involved in copper efflux *in vivo* [19,20,21].

Putative porins have also been identified in mycolic acids-containing bacteria that do not belong to the *Mycobacterium* genus. A hetero-oligomeric 87kDa cell-wall channel was identified in *Nocardia farcinica* and showed to be composed of two ~20kD Sec-secreted proteins: NfpA and NfpB. The two *nfpA* and *nfpB* genes are located next to each other in the genome and might be co-transcribed. Only the mixture of both proteins and not individual proteins display channel-forming activity *in vitro* [22]. A similar situation occurs in *Corynebacterium glutamicum* where the two small proteins, PorA and PorH, need to be associated in order to form a functional channel in the mycomembrane [23,24]. These proteins, the genes of which are co-transcribed, are 45 and 57 residues-long respectively, and devoid of any characterized signal sequence [25]. The tridimensional structure of the PorHA channel is unknown, nor the mechanism of PorA/PorH secretion through the inner membrane and targeting to the mycomembrane. Deletion of *porA* causes a small growth defect and an increase in antibiotic resistance, in agreement with a porin function *in vivo* [25].

In addition to the cation-selective PorHA channel, an anion-selective porin, PorB, was identified in *C*. *glutamicum*. Indications for an *in vivo* function of PorB came from the observation that growth of a CgΔ*porA* mutant strain is impaired in the presence of citrate, a compound that blocks PorB *in vitro* porin activity [26]. This result suggests that a functional PorB pore is required for optimal growth in the absence of the PorHA channel. The *porB* gene is co-transcribed with *porC*, a gene encoding a protein 30% identical to PorB. PorB and PorC are 126 and 123 aa-long respectively and each possesses a putative Sec-dependant signal sequence. Although an *in vitro* pore-forming activity was measured for PorB, such an activity was not demonstrated yet for PorC [26]. Recombinant PorB was expressed and purified from *E*.*coli* and its X-ray structure solved at 1.8Å resolution. It folds as a compact domain made of four α-helices tied together by a disulfide bridge, suggesting that the channel would be formed by the oligomerization of several PorB sub-units [27].

In addition to their small size and putative oligomeric state, *Corynebacterium* porins also differentiate themselves from Gram-negative bacteria porins by a Mycolata-specific post-translational modification. Indeed, PorH and PorA, together with another small protein of unknown identity and function (ProtX) are modified by the covalent attachment of mycolic acids to serine residues [6, 7, 28]. The site of modification of PorA and PorH were determined as S15 and Ser56, respectively [6,7]. Importantly, reconstitution studies found that the PorHA oligomer channel activity was dependent on PorA mycoloylation, whereas PorH modification was dispensable [24].

How mycolic acids are transfered onto proteins is still not clearly established. A family of proteins, found in the *Corynebacteriales* envelopes, is known to transfer mycolic acid residues onto various acceptors, including trehalose monomycolate (TMM) (to form trehalose dimycolate (TDM)) and the terminal end of arabinogalactan (to form the mycolyl-arabinogalactan peptidoglycan complex). *C*. *glutamicum* encodes six of these "mycoloyltransferases" (Myts) [29]. Among these six, only MytC was found to be necessary for protein mycoloylation. Indeed, in a Δ*mytC*::*Km* mutant, PorA, PorH and ProtX are not modified anymore. In this strain, accumulation of TMM was observed, suggesting that MytC uses TMM as the mycolate donor for proteins modification [30]. Still, it is not yet known when, during the biogenesis of mycoloylated proteins and where, in the envelope, the mycolate transfer is taking place. In addition, the exact role of protein mycoloylation is still unknown and is particularly interesting to study, as it does not substitute for the classical lipoproteins pathway, which also occurs in *C*. *glutamicum* [31]. Finally, the generality of the mycoloylation process still needs to be defined. Indeed, targets for mycoloylation other than PorA, PorH and ProtX have not been described so far in *C*. *glutamicum* and whether mycoloylation is restricted to Corynebacteria or also exists in other *Corynebacteriales* remains to be addressed. A key to understand the mechanisms and functional roles of mycoloylation is hence to identify its substrates and the corresponding modifications sites.

Several methods exist in order to assess whether a protein is acylated or not. Radioactive labeling has been extensively used for this purpose but is cumbersome to handle. Mass spectrometry (MS) can be used to determine whether a protein is modified or not and will give important information on the mass of the added group. However, not all protein will give a proper signal in MS, and, as for radioactive labeling, this method can only be used to characterize a known protein but is not suitable for large-scale identification of acylated proteins. Bioinformatic approaches, which rely on the search of a recognizable motif in protein sequences, cannot be applied for the global search of new mycoloylated proteins, as only 2 non-conserved sites of modification have been identified so far. In contrast, metabolic labeling has proved useful in the isolation and discovery of new lipidated proteins. This method relies on alkyne or azido-tagged analogues of the natural substrate fatty acids, which can be used by the cellular machineries to modify target proteins. Once the probes have been incorporated into lipidated proteins, they can be further linked to detection tags or affinity handles by specific and efficient “click-chemistry” reactions [32]. This approach has been applied to large-scale profiling of palmitoylated proteins in eukaryotes [33–35], but rarely so far in the detection of protein lipidation in prokaryotes [36,37,38]. In contrast, metabolic labeling has been successfully used in *Corynebacteriales* to label non-proteinaceous components of the mycomembrane (trehalose monomycolate and trehalose dimycolate), using trehalose analogues [39, 40].

Our goal here was to test whether metabolic labeling could be used in order to label mycoloylated proteins. We show that an alkynyl-fatty acid can indeed be incorporated into mycolic acids-modified proteins as specific labeling of PorA, PorH and ProtX could be observed following click-chemistry. The labeling was dependent on both the presence of MytC and the synthesis of mycolic acids, indicating that it indeed represents protein mycoloylation. This approach was further used to determine ProtX mycoloylation site. Finally, we present evidences that two other porins form *C*. *glutamicum*, PorB and PorC, are also mycoloylated.

## Materials and methods

### Bacterial strains and crowth conditions

All *C*. *glutamicum* strains used in this study are derivative of the strain ATCC 13032 RES167 [41]. *C*. *glutamicum* ATCC13032 RES167 and *Cg*Δ*porHporA* [42] were grown in brain heart infusion (BHI) medium with shaking (220 rpm) at 30°C. The *C*. *glutamicum* mutant strains Δ*pks13*::*km* [43], Δ*mytC*::*km* [30] and Δ*cg2875*::*km* (this study, see below) were grown in BHI medium containing kanamycin at a final concentration of 25 μg/ml. Chloramphenicol, at a final concentration of 10 μg/ml, was added to the medium when the strains transformed with derivatives of the pXMJ19 cloning vector were cultivated. Transformation of *C*. *glutamicum* was performed by electroporation as described by Bonamy *et al*. (1990) [44]. All cloning steps were done in *Escherichia coli* DH5α grown at 37°C in Luria-Bertani (LB) medium supplemented with appropriate antibiotics.

Construction of the *C*. *glutamicum* Δ*cg2875*::*km* mutant was performed by allelic replacement of the *cg2875* gene by a Km^R^ cassette as described previously [30]. Briefly, two DNA fragments (0.6 kb each) flanking the *cg2875* gene at its 5’ and 3’ extremities were amplified by PCR from *C*. *glutamicum* genomic DNA using primers protXdel1 + protXdel2 and protXdel3 + protXdel4, respectively (Table 1). Then, the 2 fragments were inserted in the plasmid pMCS5 (MoBiTec, Göttingen, Germany) and a kanamycin resistance cassette was inserted between them to give pMCS5::*cg2875*. This plasmid was transferred into *C*. *glutamicum* by electroporation, and transformants were selected on plates containing kanamycin. Transformants in which allelic replacement had occurred between the WT chromosomal *cg2875* gene and the mutated plasmid-borne allele were verified by PCR using primers ver1 and ver2 (Table 1). One strain, *C*. *glutamicum* Δ*cg2875*::*km*, was selected for further studies.

**Table 1:** Oligonucleotides used in this study.

Primer	Sequence (5’-3’)
ProtXDel1	TATCT*GGATCC*AGGACCTTATCC
ProtXDel2	AATA*CCGCGG*TGTGATCTCTCTTTC
ProtXDel3	TTAT*CCGCGG*TCGCGATCCTTC
ProtXDel4	GGG*AAGCTT*CTTGCACACCATTG
Ver1	AAATGGCCCAGTTTTTGACG
Ver2	TGGTCAGTGTCCGTCTCAAG
Dir-pXMJ19	GGATAACAATTTCACACAGG
Rev-pXMJ19	GGCCCAGTCTTTCGACTGAGCC
DirPorA-S15V	CTTGATGTCCTT**GT**CGGCTCCGGC
RevPorA-S15V	GCCGGAGCCG**AC**AAGGACATCAAG
DirProtX-XbaI	G*CTCAGA*CCAGTCAGACAGCAAGAG
RevProtXhis-EcoRI	GC*GAATTC*TTACTAATGGTGATGGTGATGGTGGGAAGAAGCGGTAACG
DirProtX-S3A	GATCACAATGACC**G**CTGTATTCG
RevProtX-S3A	CGAATACAG**C**GGTCATTGTGATC
DirProtX-S10A	CGATATCATCCAG**G**CCATCTTCG
RevProtX-S10A	CGAAGATGG**C**CTGGATGATATCG
DirProtX-S21V	CCTCGTTGGC**GT**CATCTTCGCTG
RevProtX-S21V	CAGCGAAGATG**AC**GCCAACGAGG
DirProtX-S32A	GGGCGTCTTCGAC**G**CCATCGTTACCGC
RevProtX-S32A	GCGGTAACGATGG**C**GTCGAAGACGCCC
DirProtX-S37A	CCATCGTTACCGCT**G**CTTCCCACCATCACC
RevProtX-S37A	GGTGATGGTGGGAAG**C**AGCGGTAACGATGG
DirProtX-S38A	CCATCGTTACCGCTTCT**G**CCCACCATCACC
RevProtX-S38A	GGTGATGGTGGG**C**AGAAGCGGTAACGATGG
DirPorB-XbaI	GCG*TCTAGA*GGTGAGTTATTCATATTACCC
RevPorBhis-EcoRI	GCC*GAATTC*TTATTAGTGATGGTGATGGTGATGGGAAGAGAAGTTGGAGG
DirPorC-XbaI	GCG*TCTAGA*GCCATATTTATTTCATTTCC
RevPorChis-EcoRI	CGC*GAATTC*TTATTAGTGATGGTGATGGTGATGAGCAGTGAAGAAGGAAG

Restriction sites used for cloning are indicated in italic. Mutated bases are in bold in mutagenic primers.

### Plasmids construction

Plasmids pXMJ19-PorA_Chis_, pXMJ19-PorH_Chis_ and pXMJ19-PorH_Chis_-S56A were previously described [7,24]. pXMJ19-PorA_Chis_-S15V was constructed using the overlap PCR method [45], with pXMJ19-PorA_Chis_ as a template, Dir-pXMJ19 and Rev-pXMJ19 as flanking primers and DirPorA-S15V and RevPorA-S15V as mutagenic oligonucleotides (Table 1). To construct pXMJ19-ProtX_his_, the *cg2875* gene was amplified with DirProtX-XbaI and RevProtXhis-EcoRI (Table 1) using ATCC13032 RES167 genomic DNA as template. The 201bp PCR fragment obtained was then cloned between the XbaI and EcoRI sites of pXMJ19. Site-directed mutagenesis of ProtX serine residues was performed by the overlap PCR method using pXMJ19-ProtX_his_ as template, Dir-pXMJ19 and Rev-pXMJ19 as flanking primers and the mutagenic primers listed in Table 1.

In order to construct pXMJ19-PorB_his_ and pXMJ19-PorC_his_, the genes encoding PorB (*cg1109*) and PorC (*cg1108*), were amplified with primers DirPorB-XbaI and RevPorB_his_-EcoRI and DirPorC-XbaI and RevPorC_his_-EcoRI (Table 1), respectively. The PCR fragments (458bp and 476bp respectively) were then inserted between the XbaI and EcoRI sites of pXMJ19.

### Protein over-expression and purification

Overnight culture of *C*. *glutamicum* strains harboring the different pXMJ19-derived plasmids were diluted in 300ml fresh BHI containing appropriate antibiotics to an OD_650nm_ of 0.2 and grown until OD_650nm_ reach 3–4. Then, 1mM IPTG (isopropyl β-D-1-thiogalactopyranoside) was added to the medium and cells were grown overnight. When indicated, 20μM 17-ODYA (17-octadecynoic acid, Cayman Chemical) was added simultaneously with IPTG. The next day, cultures were harvested by centrifugation at 4 500 x g for 10 min at 4°C. The culture supernatant was re-centrifuged for 10 min at 4 500 x g at 4°C, filtrated on a 0,2μm membrane and dialyzed against 25mM phosphate buffer pH8.0. Cell pellet were washed with 300ml of 50mM Tris-HCl, pH 8.0, resuspended in 15ml 25mM phosphate buffer, pH 8.0 containing 1% LDAO (*N*,*N*-Dimethyldodecylamine *N*-oxide) and 0,1mg/ml AEBSF (4-(2-Aminoethyl) benzenesulfonyl fluoride hydrochloride) and left at 4°C for 2h with continuous shaking. The detergent extract, enriched with cell wall proteins, was collected by centrifugation at 4 000 x g for 10 min at 4°C. It was then diluted in 25 mM phosphate buffer (pH 8.0) to reach a LDAO final concentration of 0.4%, supplemented with 10mM imidazol and added to 0,5ml of Ni-NTA resin (Macherey-Nalgen) pre-washed with 25mM phosphate buffer, pH 8.0, LDAO 0,4%, 10mM imidazol. Dialyzed supernatants, supplemented with 10mM imidazol, were similarly added to 0,5ml of Ni-NTA resin pre-washed with 25mM phosphate buffer, pH 8.0, 10mM imidazol, LDAO 0,4%. After 2 hours incubation at 4°C, the resin was washed twice with 10ml 25mM phosphate buffer, LDAO 0,4%, 10mM imidazol. Proteins were eluted in 25mM phosphate buffer, LDAO 0,4%, containing 250 mM imidazole. The purity and homogeneity of eluted protein fractions were analyzed on 16% Tricine SDS–PAGE [46].

### CuAAC reaction and biotin detection

The elution fractions containing pure proteins were pooled and dialyzed against 50mM triethanolamine (TEA), pH8.0, LDAO 0,4%, 150mM NaCl. Proteins concentrations were quantified according to their molar extinction coefficient by measuring UV absorbance at 280nm (PorA_his_: 8480 cm^-1^ M^-1^; PorH_his_: 1490 cm^**-**1^ M^**-**1^; PorB_his_: 21095 cm^**-**1^ M^**-**1^; PorC_his_: 3105 cm^**-**1^ M^**-**1^). Concentration of ProtX_his_, which lacks tryptophan and tyrosine residues, was estimated on gel. Purified protein (5–20μg) were brought up to 51μl in 50mM TEA, 150mM NaCl, 0,4% LDAO. To this, 6μl of a master mix (with or without azido-biotin) and 3μl of 20% SDS were added. The master mix is composed of 0.1mM azido-PEG3-biotin (Az-Biot; from a 50mM stock solution in DMSO, Sigma), 1mM TCEP (tris(2-carboxyethyl)phosphine hydrochloride, from a 50mM freshly prepared solution in ddH2O), 0.1mM TBTA (tris[(1-benzyl-1H-1,2,3-triazol-4-yl)methyl]amine, from a 2mM stock solution in 4:1 tert-butanol:DMSO) and 1mM CuSO_4_-5H_2_O (from a 50mM freshly prepared solution in ddH_2_O). The reactions were incubated at 25°C for 2 hours with constant shaking (300 rpm). Then, the proteins were precipitated with chloroform/methanol. Briefly, methanol (4X the CuAAC reaction volume) was added to the reaction and thoroughly vortexed, followed by 1X chloroform and 3X ddH_2_O. Samples were then centrifuged at 16 000 g for 1 min and the aqueous layer was removed. Samples were then washed with 4X methanol, vortexed and centrifuged at 16 000g for 2 min. The pellets were dried, resuspended in 1 volume of sample buffer and heated at 10°C for 10min. Twenty microliters of sample were then separated on two 16% Tricine SDS–PAGE. One gel was stained with Coomassie brillant blue. The proteins from the other gel were transferred onto a 0.2μm nitrocellulose membrane. The membrane was blocked with 1% bovine serum albumin (BSA) in phosphate buffer saline (PBS) 0,05% Tween 20 for one hour at room temperature, followed by incubation with streptavidin-HRP (Streptavidin-peroxidase polymer ultrasensitive, Sigma, 1:1000 in PBS), for at least one hour at 4°C. After six ten-minutes washes in PBS 0,05% Tween 20, revelation was performed using the Biorad Clarity® reagent and the chemiluminescence detection system ImageQuant LAS500 (GE Healthcare) following manufacturer's instructions.

### Mass spectrometry analysis

To prepare chloroform/methanol extracts, cells were grown overnight in BHI medium and harvested by centrifugation. The pellet was extracted with a mixture of chloroform: methanol (1:2, v/v) overnight at room temperature. The chloroform/methanol extract was recovered by centrifugation (4 000 x g, 15min, 4°C). Proteins from the extract were precipitated by adding 9 volumes of diethyl ether to one volume of extract and incubating the mixture overnight at -20°C. After centrifugation, the supernatant was discarded and the pellet was resuspended in Tris-HCl 10mM pH8.0, LDAO 0,4%. One microliter of sample (usually diluted 1:20 in the same buffer) was mixed directly on target with 1μl of the sinapinic acid matrix solution (5mg/ml in H_2_O-CH_3_CN: 0,1% trifluoroacetic acid [1:1]).

MALDI-TOF spectra were acquired on an Axima performance mass spectrometer (Shimadzu corporation) equipped with a pulsed nitrogen laser emitting at 337 nm and an accelerating voltage of 20 kV. All spectra were acquired in the positive linear mode. External mass calibration was performed on the 3 peaks corresponding to unmodified ProtX, PorA and PorH present in the organic solvant extract of the Δ*mytC*::*Km* strain.

### Cell fractionation

Five milliliters pre-cultures of strains 13032, Δ*mytC*::*Km* or Δ*pks13*::*Km* transformed with either pXMJ19-PorB_his_ or pXMJ19-PorC_his_ were grown overnight in BHI at 30°C. The next day, the cultures were diluted in fresh BHI media to an OD_650nm_ of 0.4 and cultivated at 30°C with agitation. When indicated, recombinant proteins expression was induced by addition of 1mM IPTG at OD_650nm_ = 3–4, followed by overnight growth at 30°C. The next day, two 4ml samples of overnight culture were centrifuged at 6 000 x g for 10 min. Cell pellets were washed with 50mM Tris-HCl, pH8.0, weighted and re-suspended in 25mM phosphate buffer containing 0,46% LDAO and 0,1mg/ml AEBSF (V = 5ml buffer per g of wet cells). After incubation at 4°C for 2h with continuous shaking, one sample was added to glass beads and vortexed for 15 min. Beads and unbroken cells were removed by centrifugation and the supernatant containing proteins was kept as the “total extract” (C) fraction. The other sample was centrifuged at 6000 x g for 10 min and the supernatant, corresponding to the “cell wall” (CW) fraction collected. The culture supernatants were re-centrifuged once and precipitated with 10% trichloroacetic acid (TCA) at 4°C for 30 min. The TCA precipitated proteins were then recovered by centrifugation (13 000 x g, 10 min), washed with cold acetone and resuspended in the same volume (V) of buffer.

Protein samples were then separated on 16% Tricine SDS-Page or 12% Tris-Glycine SDS-Page gels for immunobloting with polyclonal rabbit anti-CgPorB, anti-CgPorC [47], anti-CgAcn [48], or anti-CgMytA antibodies [49]. Detection was performed using an anti-rabbit-HRP conjugate (Sigma), the Biorad Clarity® reagent and the chemiluminescence detection system ImageQuant LAS500 (GE Healthcare) following manufacturer's instructions.

### Alkaline treatment

The purified PorC_his_ protein, in TEA 50mM, pH 8.0, NaCl 150mM, LDAO 0,4% was treated with 0.1 M NaOH at 30°C for the time indicated. The reaction mixture was then neutralized with stoichiometric quantities of acetic acid, before being mixed with sample buffer, heated at 100°C for 10 min and loaded on a Tricine SDS-Page gel that was then colored with Coomassie brilliant blue.

## Results

### PorA and PorH metabolic labeling

**Fig 1:**
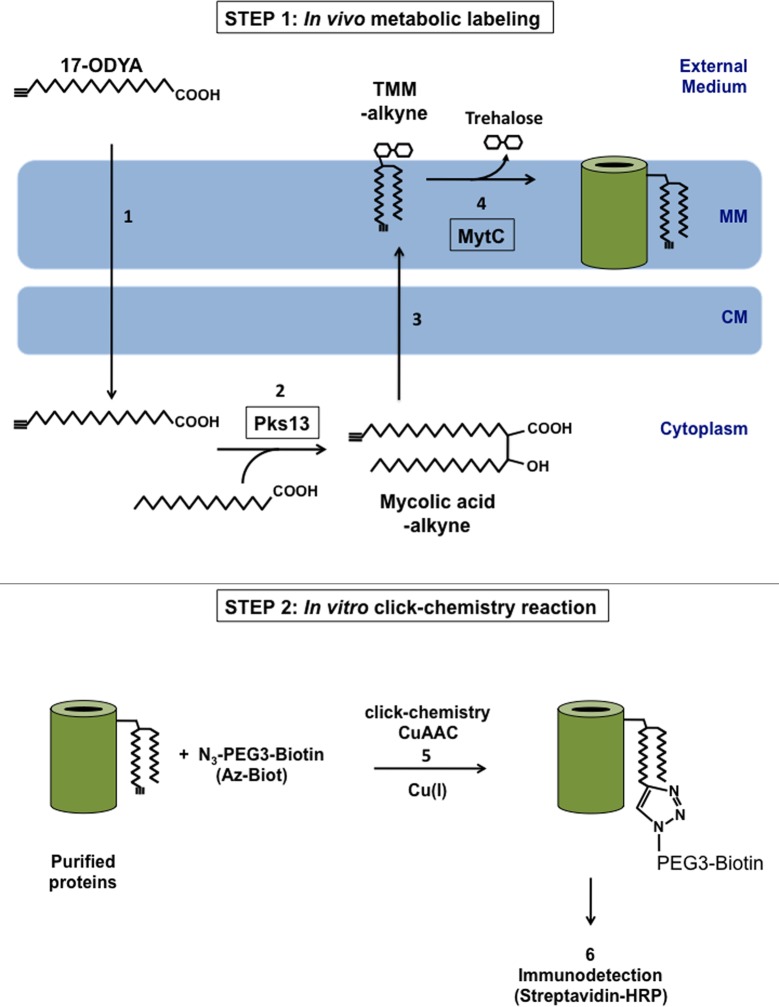
Bioorthogonal labeling of mycoloylated proteins: Principle. When added to the growth medium, a fatty-acid alkyne probe (17-ODYA), can be used by the cell to synthesize alkyne-modified mycolic acids. Specifically, mycolic acids are generated in the cytoplasm, by the condensation of two activated fatty acids, a reaction mediated by the polyketide synthase Pks13 [43]. Mycolic acids are then converted to trehalose monomycolate and transported to the envelope, where they are probably used as mycolate donor by MytC to modify a specific subset of proteins. Proteins that have incorporated an alkyne group can then be identified by Cu(I) catalyzed Azide-alkyne cycloaddition (CuAAC). In the presence of Cu(I), the azido group of azido-biotin reacts with the alkyne group, forming a triazole ring and resulting in the covalent attachment of biotin to the protein, which allows further detection of the labeled protein by streptavidin-HRP. CM: cytoplasmic membrane; MM: mycomembrane, CuAAC; Cu(I)-catalyzed azide-alkyne cycloaddition.

First, we decided to test whether metabolic-labeling and click-chemistry allow detection of the two known mycoloylated proteins, namely PorA and PorH. The accumulation of trehalose monomycolate (TMM) in the Δ*mytC*::*Km* strain and the use of TMM as substrate by other mycoloyltransferases, suggests that the mycolate donor for protein mycoloylation could be TMM [29, 30]. Although clickable analogues of trehalose can be used to generate azido-trehalose monomycolates (Az-TMM) *in vivo*, [40], these probes cannot be used here since the clickable group, located on the trehalose moiety of Az-TMM, will not be transfered onto mycoloylated proteins. Unfortunately, alkynyl or azido-TMM analogues carrying a clickable group on the fatty acid chain had not been described at the initiation of this study. In contrast, alkynyl-fatty acids analogues were available and have been shown to be incorporated in *M*. *smegmatis* proteins [36]. In addition, a previous study demonstrated that *C*. *glutamicum* could use externally added fatty acids for the production of mycolic acids [50]. Thus, we tested 17-octadecynoic acid (17-ODYA), a C18 unsaturated alkynyl fatty acid analogue, as a probe to label putative mycoloylated proteins. The reference strain ATCC 13032 of *C*. *glutamicum* was transformed with a plasmid allowing over-expression of a C-terminally His-tagged version of PorA (pXMJ19-PorA_his_ [24]) and the transformed strain cultivated in BHI until it reached an OD_650nm_ = 3–4. At this point, 1mM IPTG was added to induce expression of PorA_his_. Simultaneously, 20μM 17-ODYA were added to one culture while the other was left untreated. After overnight growth, cell wall proteins were extracted by incubation with detergent and His-tagged PorA was purified on Ni^2+^-NTA agarose. The purified protein was then reacted with azido-PEG3-biotin (Az-Biot) via a Cu(I)-catalyzed azide-alkyne cycloaddition (CuAAC) (Fig 1) [51]. Samples were separated by gel electrophoresis, transferred onto a nitrocellulose membrane and the presence of biotinylated proteins detected with a Streptavidin-Horse Radish Peroxidase (Strep-HRP) conjugate. In the cases where the protein had incorporated an alkyne group, this group should react with the azido function of an azido-PEG3-biotin conjugate (Az-Biot), resulting in the covalent linkage between the protein of interest and Biotin. The biotinylated protein can then be detected by a streptavidin-HRP conjugate (Fig 1).

**Fig 2:**
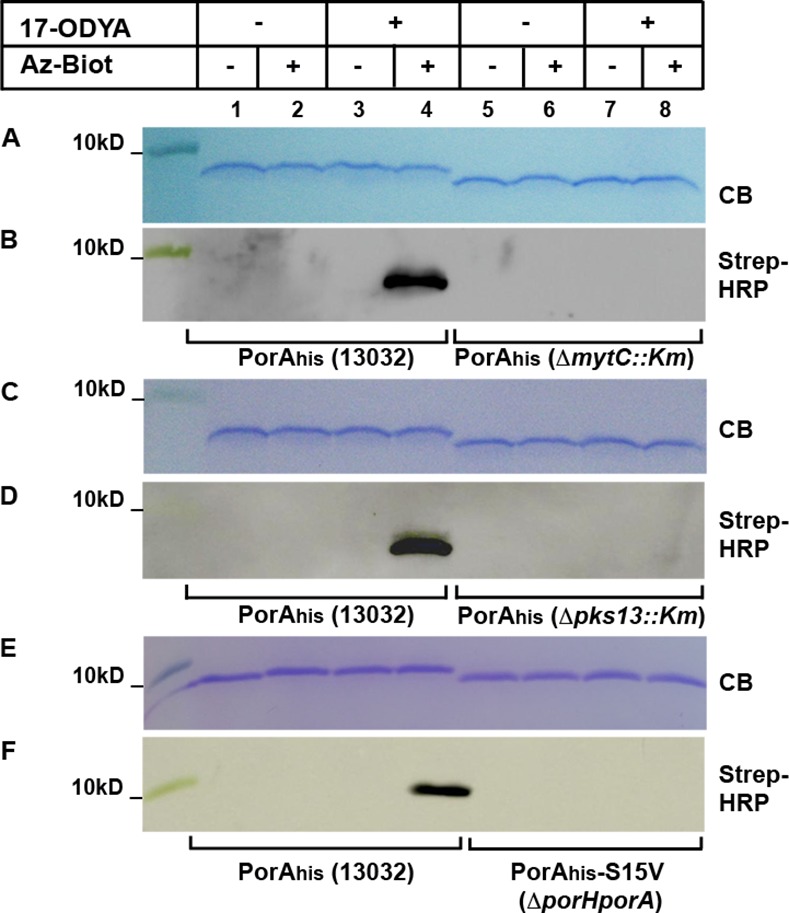
PorA metabolic labeling. *C*. *glutamicum* strains 13032, Δ*mytC*::*Km*, Δ*pks13*::*Km* or CgΔ*porHporA* over-expressing PorA_his_ were grown in the absence (lanes 1–2, 5–6) or in the presence (lanes 3–4, 7–8) of 17-ODYA. After purification, PorA_his_ was subjected to CuAAC in the presence (lanes 2,4,6,8) or absence (lanes 1,3,5,7) of Az-Biot. Samples were splitted and ran on two 16% Tricine-SDS-Page gels. One was colored with Coomassie blue (CB) while the other was used to transfer proteins on a nitrocellulose membrane that was then probed with a Streptavidin-HRP conjugate (Strep-HRP).

As shown in Fig 2 (panels B, D, F, lanes 4), a signal corresponding to biotinylated PorA_his_ was detected only in the case where the cells were grown in the presence of 17-ODYA and the CuAAC reaction performed in the presence of Az-Biot. No such signal was observed in the absence of either 17-ODYA in culture medium or Az-Biot in the CuAAC reaction, despite the presence of identical amount of purified PorA_his_ seen on gels stained with Coomassie blue (CB) (Fig 2, panels A-F, lanes 1–4). This indicates that an alkynyl group was indeed incorporated in PorA_his_ and that this incorporation was dependent on the presence of 17-ODYA in the culture medium. To determine whether this signal was representative of PorA_his_ mycoloylation, the same experiment was performed with PorA_his_ purified from the Δ*mytC*::*Km* mutant. This strain, in which protein mycoloylation is abolished, lacks MytC, the candidate enzyme for the transfer of mycolates onto proteins (Fig 1) [30]. When purified from the mutant strain, PorA_his_ was not recognized by the Streptavidin-HRP conjugate after CuAAC (Fig 2, panel B, lanes 5–8). This indicates that PorA_his_ was not biotinylated by CuAAC and hence that the alkynyl group had not been incorporated in the non-mycoloylated protein produced in the Δ*mytC*::*Km* genetic background. As it is clearly visible on the figure (Fig 2, panel A, lanes 5–8), when PorA_his_ was purified from this strain, it migrated faster on Tricine SDS-Page gels compared with the protein purified from the parental strain, which is most likely due to the absence of mycolate attached to the protein. Although the MytC-dependency of PorA_his_ labeling suggests that the signal indeed is representative of protein mycoloylation, we wanted to eliminate any possibility of MytC-mediated direct transfer of 17-ODYA onto PorA_his_. Thus, to verify that the signal observed in the wild-type strain corresponded to PorA_his_ modified with alkynyl-mycolic acids and not with 17-ODYA, the protein was purified from the Δ*pks13*::*Km* mutant which lacks the enzyme that catalyzes the condensation of two fatty acids to yield the mycolic motif (Fig 1) [43]. Similarly to what was observed in the Δ*mytC*::*Km* background, PorA_his_ purified from this strain showed faster migration on SDS-Page gel and was not detected by Streptavidin-HRP after CuAAC (Fig 2, panels C-D, lanes 5–8). Finally, we verified that the mutation of PorA mycoloylation site (Ser15) abolished labeling. To avoid any co-purification of endogenous, modified PorA or PorH proteins, PorA_his_-S15V was purified from the *Cg*Δ*porHporA* strain [42]. As expected, no labeling of PorA_his_-S15V was observed in the presence of 17-ODYA. Altogether, these results show that PorA_his_ labeling is dependent on the presence of MytC, the integrity of the mycoloylation site, and on mycolic acid synthesis. Thus, *C*. *glutamicum* is able to use externally added 17-ODYA to produce alkynyl mycolic acids, some of which being then transferred onto the Ser15 residue of PorA_his_ via a MytC-dependent process.

**Fig 3:**
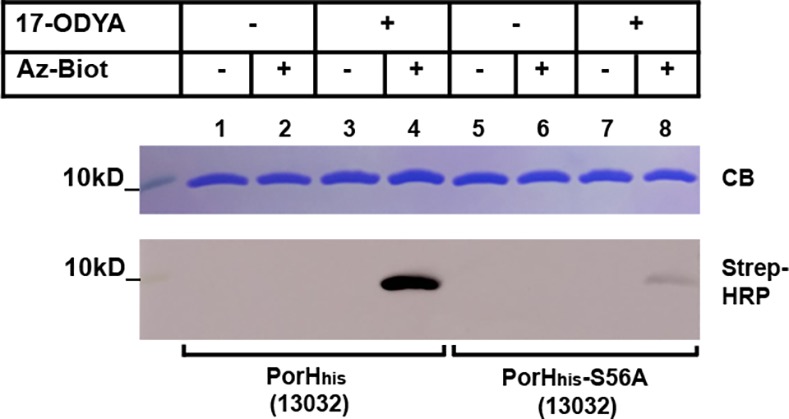
PorH metabolic labeling. *C*. *glutamicum* strains 13032 over-expressing PorH_his_ (lanes 1–4) or PorH_his_-S56A (lanes 5–8) were grown in the absence (lanes 1–2, 5–6) or in the presence (lanes 3–4, 7–8) of 17-ODYA. After purification, PorH_his_ was subjected to CuAAC in the presence (lanes 2,4,6,8) or absence (lanes 1,3,5,7) of Az-Biot. Samples were splitted and ran on two 16% Tricine-SDS-Page gels. One was colored with Coomassie blue (CB) while the other was used to transfer proteins on a nitrocellulose membrane that was then probed with a Streptavidin-HRP conjugate (Strep-HRP).

Similar experiments were then attempted with PorH_his_. Surprisingly however, PorH_his_, as well as the non-mycoloylated mutant PorH_his_-S56A, could only be expressed and purified from the wild-type strain. No protein, or very low amount of it, could be recovered form the strains Δ*mytC*::*Km*, Δ*pks13*::*Km* or CgΔ*porHporA*. The CuAAC reactions were thus performed with proteins purified from the wild-type background. As previously seen for PorA_his_, PorH_his_ was only recognized by Streptavidin-HRP when cells were grown in the presence of 17-ODYA and CuAAC performed in the presence of Az-Biot (Fig 3, lanes 1–4). Hence PorH_his_ can also be labeled with 17-ODYA. The non-mycoloylated mutant PorH_his_-S56A was found to migrate faster than PorH_his_ on gel, however the gel shift was less pronounced than between PorA_his_ and PorA_his_-S15V. Unexpectedly, a low signal was observed with PorH_his_-S56A purified from cultures grown with 17-ODYA and reacted with Az-biot (Fig 3, lane 8). Because the mycoloylation site of PorH was unambiguously determined as S56 [7], we believe that this signal originates from the chromosomally encoded PorH which, in the 13032 strain, is mycoloylated. As PorA and PorH are thought to form hetero-oligomers in the envelope [23], endogeneous PorH could potentially be co-purified with the plasmid-encoded PorH_his_-S56A and generate a signal when cells are metabolically labeled. Indeed, a similar low background labeling was also observed when CuAAC was performed on PorA_his_-S15V purified from the wild-type strain instead of the CgΔ*porHporA* strain (not shown).

### Identification of ProtX

**Fig 4:**
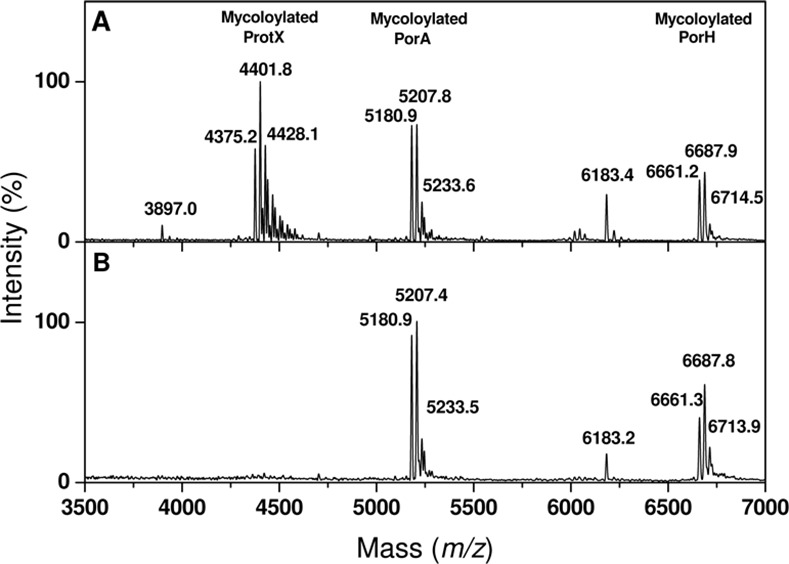
Mass spectrometry analysis of solvent-extracted proteins from wild-type and Δ*cg2875*::*Km* strains. Positive linear MALDI-TOF mass spectrum of the hydrophobic proteins extracts prepared from *C*. *glutamicum* wild-type (13032)(A) and Δ*cg2875*::*Km* (B).

Matrix-assisted laser desorption/ionization time-of-flight (MALDI-TOF) analysis of *C*. *glutamicum* cell wall extracts has previously shown that, in addition to PorA and PorH, a third protein, ProtX, was mycoloylated. However, the gene encoding ProtX was not identified [6]. The mass corresponding to ProtX when non-modified, determined in the Δ*pks13*::*Km* or Δ*mytC*::*Km* strains, is 3897 Da [6, 30]. We assumed that this mass represents a sodium adduct, as both PorA and PorH are found as Na^+^-adduct in the cell wall extracts spectra [6]. Thus, the *C*. *glutamicum* genome was mined for a gene encoding a protein of theoretical mass 3874 Da that would form a 3897 Da sodium adduct. Only one candidate fitting these criteria was found: *cg2875*. The *cg2875* gene encodes a putative protein of 38 residues that does not possess a classical signal sequence and displays no sequence homology with either PorA, PorH, or any other proteins of known function. This protein was however identified in a *C*.*glutamicum* membrane proteome analysis, indicating that it is readily expressed and present in the cell envelope [52]. In order to test whether *cg2875* indeed encodes ProtX, allelic exchange was performed to replace this gene by a kanamycin (Km)-resistance cassette. The lipophilic constituents of the Δ*cg2875*::*Km* strain were extracted with organic solvants and compared to those of the wild-type strain by MALDI-TOF mass spectrometry. As can be seen on Fig 4, a comparison of the MALDI-TOF profiles obtained from the two strains clearly showed that whereas all peaks corresponding to PorA and PorH are present in both strains, the peaks corresponding to unmodified ProtX (M+Na^+^_measured_ at *m/z* 3897.0) or ProtX modified with corynomycolic acids (M+Na^+^_measured_ at *m/z* 4375.2, 4401.8 and 4428.1), are missing in the Δ*cg2875*::*Km* strain (Fig 4 Panel B). This result hence indicates that *cg2875* indeed encodes the protein ProtX.

### ProtX metabolic labeling and determination of the site of mycoloylation

**Fig 5:**
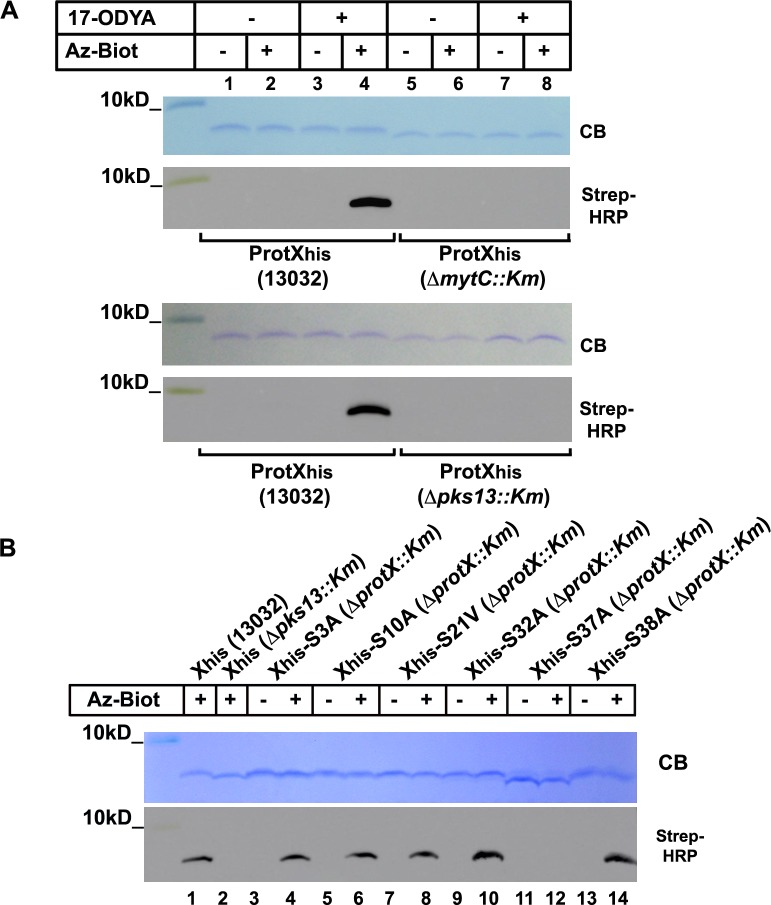
ProtX metabolic labeling and mycoloylation site determination. (A) *C*. *glutamicum* strains 13032, Δ*mytC*::*Km*, or Δ*pks13*::*Km* over-expressing ProtX_his_ were grown in the absence (lanes 1–2, 5–6) or in the presence (lanes 3–4, 7–8) of 17-ODYA. After purification, ProtX_his_ was subjected to CuAAC in the presence (lanes 2,4,6,8) or absence (lanes 1,3,5,7) of Az-Biot. Samples were splitted and ran on two 16% Tricine-SDS-Page gels. One was colored with Coomassie blue (CB) while the other was used to transfer proteins on a nitrocellulose membrane that was then probed with a Streptavidin-HRP conjugate (Strep-HRP). (B) ProtX_his_ and ProtX_his_ single serine mutants were over-expressed in 13032 and Δ*pks13*::*Km* or Δ*protX*::*Km*, respectively in the presence of 17-ODYA, purified and subjected to CuAAC in the presence (lanes 1,2,4,6,8,10,12,14) or absence (lanes 3,5,7,9,11,13) of Az-Biot. Samples were splitted and ran on two 16% Tricine-SDS-Page gels. One was colored with Coomassie blue (CB) while the other was used to transfer proteins on a nitrocellulose membrane that was then probed with a Streptavidin-HRP conjugate (Strep-HRP).

Once identified, the *protX* encoding gene was cloned with a C-terminal His-tag in the *E*.*coli/ C*.*glutamicum* pXMJ19 vector [24] under the control of the *tac* promoter. The protein was over-expressed in the strains 13032, Δ*mytC*::*Km* and Δ*pks13*::*Km* and subjected to metabolic labeling and click-chemistry as described above. As previously observed for PorA_his_ and PorH_his_, ProtX_his_ labeling was only observed when the protein was purified from the wild-type strain grown in presence of 17-ODYA and subjected to CuAAC in the presence of azido-biotin (Fig 5, panel A, lane 4). In contrast, the proteins purified from the Δ*mytC*::*Km* or Δ*pks13*::*Km* background, which migrated faster on the gel, were not detected with the streptavidin-HRP conjugate (Fig 5, panel A). Hence, the mycoloylation of ProtX can also be detected by 17-ODYA metabolic labeling which further confirm that ProtX is the product of *cg2875*. To explore other potential applications of this approach, we decided to use it in order to identify ProtX mycoloylation site. To do so, the 6 serine residues of the protein (Ser3, Ser10, Ser21, Ser32, Ser37 and Ser38) were systematically mutated to either alanine or valine and the recombinant proteins were over-expressed and purified from the Δ*cg2875*::*Km* strain (now called Δ*protX*::*Km*) grown in the presence of 17-ODYA, before being subjected to CuAAC. As observed in Fig 5 (panel B), a signal was detected for all mutants in the presence of azido-biotin, except for the ProtX_his_-S37A mutant (Fig 5, panel B, lanes 11–12). Together with the fact that ProtX_his_-S37A migrates slightly faster on Tricine-SDS-PAGE gel compared with other mutants, this result suggests that Ser37 is the site of modification of ProtX.

**Fig 6:**
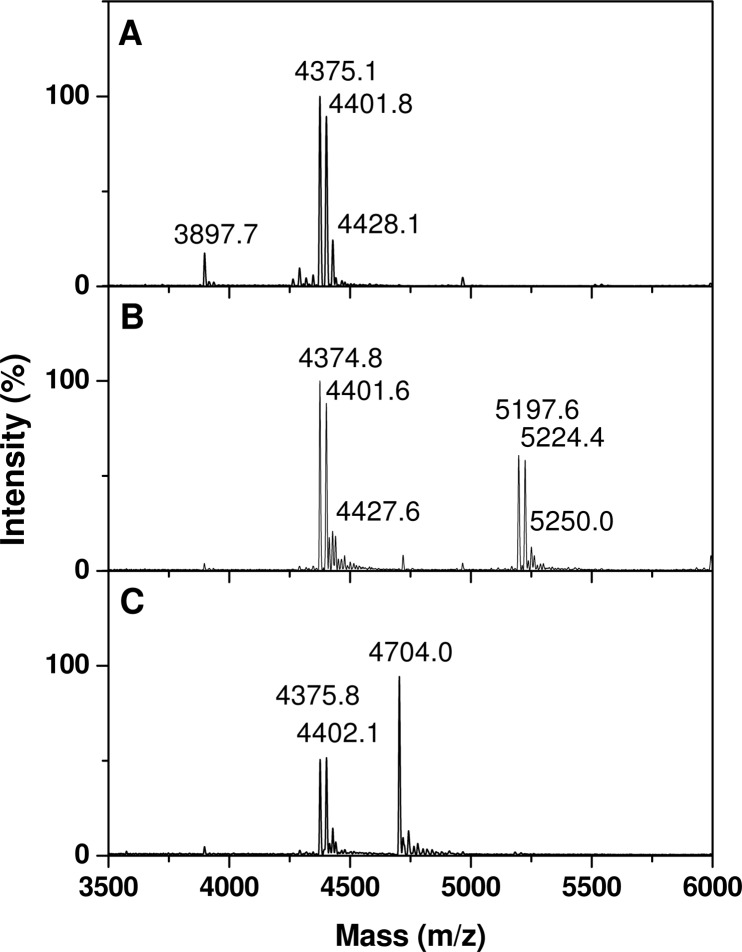
Mass spectrometry analysis of solvent-extracted proteins from CgΔ*porHporA* strain over-expressing ProtX_his_ or ProtX_his-_S37A. Positive linear MALDI-TOF spectra of the hydrophobic proteins extracts from CgΔ*porHporA* (A), CgΔ*porHporA* transformed with pXMJ19-ProtX_his_ and grown in the presence of 1mM IPTG (B) or CgΔ*porHporA* transformed with pXMJ19-ProtX_his-_S37A and grown in the presence of 1mM IPTG (C).

To confirm this result, the *in vivo* mycoloylation status of ProtX_his_-S37A was determined by mass spectrometry. Because the theoretical masses of chromosomally encoded PorA mycoloylated with either a C32:0 mycolic acid (M+Na^+^ at *m/z* 5181.35), a C34:1 mycolic acid (M+Na^+^ at *m/z* 5207.35); or a C36:2 mycolic acid M+Na^+^ at *m/z* 5233.35)) are very close from the expected masses for mycoloylated ProtX_his_-S37A (M+Na^+^ at *m/z* 5182.23, 5208.23 or 5234.23, if modified with a C32:0, C34:1 or C36:2, respectively), the plasmids encoding ProtX_his_ and ProtX_his_-S37A were transformed in the CgΔ*porHporA* strain [42]. The transformants obtained were then grown in the presence of 1mM IPTG to induce expression of ProtX_his_ and ProtX_his_-S37A. Organic solvants extracts of these cells were prepared and compared to the one obtained with the untransformed CgΔ*porHporA* strain by MALDI-TOF analysis. As expected, in the untransformed CgΔ*porHporA* strain, no peaks corresponding to endogeneous PorA or PorH were observed. Instead, peaks corresponding to the chromosomally encoded ProtX were observed: a small peak at *m/z* 3897.7 corresponding to the pseudomolecular mass (M+Na^+^) of the non-mycoloylated form and a major series of M+Na^+^_measured_ peaks at *m/z* 4375.1; 4401.8 and 4428.1 for the protein modified with a C32:0; C34:1 or C36:2, respectively (Fig 6A). When the recombinant ProtX_his_ was over-expressed in the same strain, a triplet of peaks appeared at *m/z* 5197.6; 5224.4 and 5250.0, which corresponded to the masses of ProtX_his_ to which is added either 478, 504 or 530Da (Fig 6B). These masses correspond well with the expected masses of the 3 major corynomycolic acid species naturally occurring in *C*. *glutamicum* (C32:0, C34:1, and C36:2) and hence confirm that the recombinant ProtX_his_ is mycoloylated when expressed in the CgΔ*porHporA* strain. In contrast, when ProtX_his_-S37A was over-expressed in the same strain, only a single peak appeared at *m/z* 4704.0, which corresponded to the expected mass for unmodified ProtX_his_-S37A (M+Na^+^_expected_ = 4704.23). These results hence confirm that the serine 37 is the mycoloylation site of ProtX.

### Evidence for PorB and PorC mycoloylation in *C*. *glutamicum*

**Fig 7:**
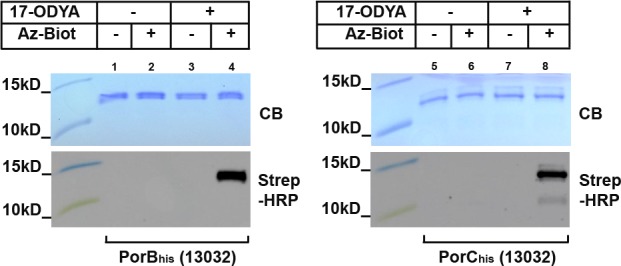
PorB and PorC metabolic labeling. *C*. *glutamicum* strains 13032 over-expressing PorB_his_ (lanes 1–4) or PorC_his_ (lanes 5–8) were grown in the absence (lanes 1–2, 5–6) or in the presence (lanes 3–4, 7–8) of 17-ODYA. After purification, PorB_his_ and PorC_his_ were subjected to CuAAC in the presence (lanes 2,4,6,8) or absence (lanes 1,3,5,7) of Az-Biot. Samples were splitted and ran on two 16% Tricine-SDS-Page gels. One was colored with Coomassie blue (CB) while the other was used to transfer proteins on a nitrocellulose membrane that was then probed with a Streptavidin-HRP conjugate (Strep-HRP).

Of the 3 mycoloylated proteins identified so far, two of them (PorA and PorH) together form outer membrane channels, the *in vitro* activity of which is dependent on mycoloylation [24]. We thus wondered if mycoloylation could be found in other *C*. *glutamicum* pore-forming proteins. In this bacterium, in addition to PorHA, only one other porin has been described to date, the anion selective, probably oligomeric, channel-forming protein PorB. We hence cloned *porB* and *porC* in the pXMJ19 vector with a C-terminal His-tag. The recombinant proteins were over-expressed and purified from the envelope of the *C*. *glutamicum* 13032 strain cultivated in the presence or absence of 17-ODYA and the purified proteins reacted with Az-Biot, as previously described. As can be seen on Fig 7, a signal corresponding to biotinylated PorB_his_ and PorC_his_ was detected only in the cases where the cells were grown in the presence of 17-ODYA and the CuAAC reaction performed in the presence of Az-Biot. This result indicates that an alkynyl group was incorporated in PorB_his_ and PorC_his_ and represents the first evidence of a potential lipidation of PorB or PorC in *C*. *glutamicum*. Because nor PorB or PorC possess a lipobox and because, according to PorB structure, the 2 cysteines present in PorB are involved in the formation of a disulfide bridge [27], it is highly unlikely that these proteins are *S*-acylated. Instead, they are most probably mycoloylated. If this is the case, it is expected that the 17-ODYA labeling would be abolished in the Cg Δ*mytC*::*Km* or Δ*pks13*::*Km* strains.

### PorB and PorC mycoloylation is important for cell envelope association

**Fig 8:**
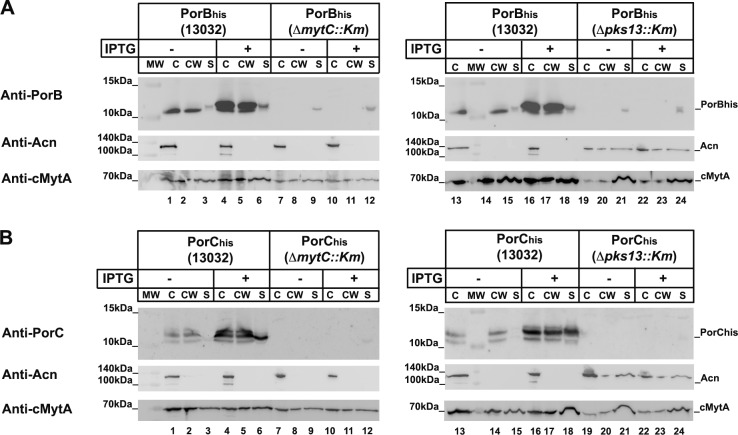
PorB and PorC expression and localization. *C*. *glutamicum* strains 13032, Δ*mytC*::*Km*, or Δ*pks13*::*Km* transformed with either pXMJ19-PorB_his_ (A) or pXMJ19-PorC_his_ (B) were grown overnight in the absence or in the presence of IPTG. Total cell extract (C), cell wall fraction (CW) and supernatant (S) were separated on Tricine-SDS-Page or Tris-Glycine-SDS-Page gels and immunoblotted with anti-PorB (A) or anti-PorC (B) antisera. The same samples were also probed with anti-aconitase (Acn, cytoplasmic marker) and anti-cMytA (envelope and supernatant marker).

Hence, in order to insure that PorB and PorC labeling is indeed representative of a modification by mycoloylation, we then aimed at testing the labeling of proteins purified from the Cg Δ*mytC*::*Km* and Δ*pks13*::*Km* strains. To first verify whether the proteins were expressed in these strains, total cell extracts (C), cell wall fractions (CW) and supernatants (S) were prepared and analyzed by immuno-blotting using anti-PorB or anti-PorC antisera. In the parental strain (13032) transformed with pXMJ19-PorB_his_ and grown in the absence of IPTG, a band corresponding to endogenous PorB was detected in all 3 fractions, with the majority of the protein, however, being located in the envelop (Fig 8A, anti-PorB, lanes 1–3). Upon IPTG induction, an increased amount of protein, migrating slightly above endogenous PorB, and corresponding to PorB_his_, was observed in all fractions (Fig 8A, anti-PorB, lanes 4–6). This indicates that PorB is mainly located in the envelope, although a fraction of the protein is released in the culture supernatant, especially upon over-expression. Interestingly, in the Δ*mytC*::*Km* and Δ*pks13*::*Km* strains, the overall level of PorB_his_ was decreased and the protein was only detected in the culture filtrate (Fig 8A, anti-PorB, lanes 7–12 and 19–24). To ensure that PorB_his_ external localization was not due to cell lysis, the same samples were probed with antibodies directed against the cytoplasmic protein acotinase (Acn) [48]. As can be seen on Fig 8A, aconitase was only detected in the total cell extract (C) of the ATCC 13032 and Δ*mytC*::*Km* strains, indicating that cell integrity was not compromised in these strains (Fig 8A, anti-Acn, lanes 1,4,7,10). In the Δ*pks13*::*Km* strain however, Acn was detected not only in the total cell extract, but also in the cell wall and supernatant fractions (Fig 8A, lanes 19–24), suggesting a profound change of the cell envelope in this strain, induced by the lack of mycolic acid residues. Importantly, although previous studies indeed showed that the total absence of mycolic acids results in an altered envelope, the exact consequences on cell permeability, including an eventual increased in the permeability of the cytoplasmic membrane, were not reported [9, 43]. As a control for mycomembrane integrity, we probed the different fractions with anti-cMytA antibodies for the presence of the mycoloyltransferase cMytA, an enzyme that is both associated to the mycomembrane and secreted in the medium [53, 54]. As expected, cMytA was equally distributed between the cell wall and supernatant fractions in the wild-type strain. Similar results were obtained for the Δ*mytC*::*Km* strain, hence confirming that PorB_his_ secretion, in this strain, is not due to a lack of envelop integrity (Fig 8A, anti-cMytA; lanes1-6; 13–18). The Δ*pks13*::*Km* strain however, showed again some signs of envelop pertubation, with the level of cMytA increased in the supernatant fraction (Fig 8A, anti-cMytA, lanes 19–24).

When the same experiments were performed with PorC_his_, we found that in the ATCC13032 strain, PorC_his_ is already expressed even in the absence of IPTG and can be detected, together with the endogenous PorC, in all fractions. However, like PorB, it is most abundant in the cell wall (Fig 8B, anti-PorC, lanes 1–3 and 13–15). Upon induction with IPTG, the level of PorC_his_ increases in all fractions, especially in the supernatant (Fig 8B, anti-PorC, lanes 4–6). As observed for PorB_his_, in the Δ*mytC*::*Km* and Δ*pks13*::*Km* strains, the level of PorC decreased drastically with, only a very weak, diffuse signal observed in the supernatant in the presence of IPTG (Fig 8B, anti-PorC, lanes 12 and 24). The fractionation controls confirm that this localisation is relevant in the case of the Δ*mytC*::*Km* strain and is not the consequence of a general envelope perturbation.

In conclusion, the lack of MytC and Pks13 both led to a decrease in PorB_his_ and PorC_his_ expression, together with the total release of the proteins in the culture medium. Although in the Δ*pk13*::*Km* strain, this localisation cannot be interpreted because of a compromised envelop integrity, in the Δ*mytC*::*Km* strain, it appears to be a direct consequence of the lack of protein mycoloylation, as it does not affect other mycomembrane protein such as cMytA.

Hence, MytC-mediated mycoloylation appears to be important for proper targeting and/or insertion of PorB and PorC to the mycomembrane.

**Fig 9:**
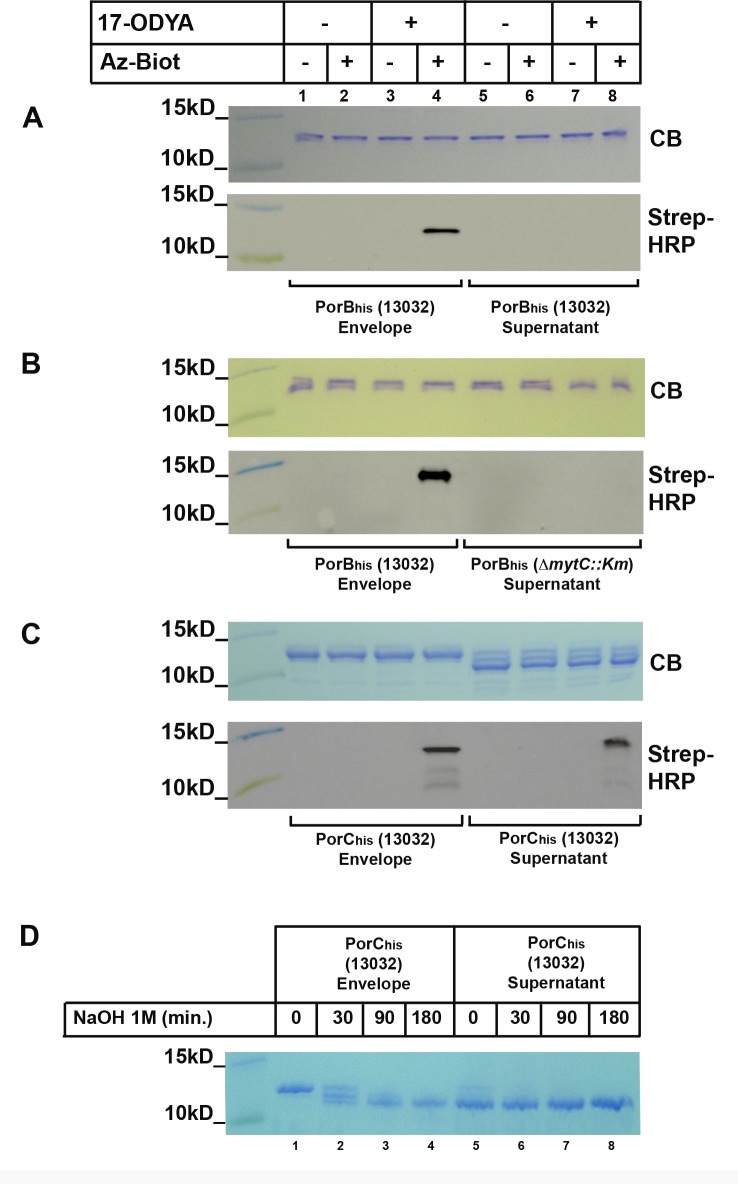
Cell wall and secreted PorB and PorC metabolic labeling and NaOH treatment. *C*. *glutamicum* strains 13032 or Δ*mytC*::*Km* over-expressing PorB_his_ (A, B) or PorC_his_ (C) were grown in the absence (lanes 1–2, 5–6) or in the presence (lanes 3–4, 7–8) of 17-ODYA. After purification from either the envelope or the culture filtrate, the proteins were subjected to CuAAC in the presence (lanes 2,4,6,8) or absence (lanes 1,3,5,7) of Az-Biot. Samples were splitted and ran on two 16% Tricine-SDS-Page gels. One was colored with Coomassie blue (CB) while the other was used to transfer proteins on a nitrocellulose membrane that was then probed with a Streptavidin-HRP conjugate (Strep-HRP). (D) PorC_his_ purified from either the envelope or the supernatant of 13032 was treated with NaOH 0,1M for the indicated time. The reaction was stopped by addition of an equimolar amount of acetic acid. Samples were then splitted and ran on a 16% Tricine-SDS-Page gel and stained with Coomassie brilliant blue.

We then purified PorB_his_ from the envelope and the supernatant of the 13032 strain and from the culture supernatant of the Δ*mytC*::*Km* strain grown in the presence of 17-ODYA and subjected the purified proteins to CuAAC. As previously observed in Fig 7, PorB_his_ purified from the envelope of the wild-type strain gave a signal when reacted with azido-biotin (Fig 9, panels A and B, lanes 1–4). In contrast, when the protein was purified from the supernatant of the same strain it appears not to be modified (Fig 9, panel A, lanes 5–8). This indicates that only the modified form of the protein is able to remain associated with the bacterial envelope and thus suggests that mycoloylation is important for this association. In addition, the same protein purified from the supernatant of the Δ*mytC*::*Km* strain did not show any labeling either (Fig 9, panel B, lanes 5–8), thus implying that in the absence of MytC, the protein is not mycoloylated, cannot remain associated with the envelope, and is hence released in the culture medium.

We then metabolically labeled PorC_his_ and purified the protein from the envelope and supernatant of the wild-type strain. Analysis of purified proteins on SDS-PAGE gels revealed that, in contrast with PorC_his_ isolated from the envelop, which migrates as a single major band, the secreted fraction of PorC_his_ migrates as three bands: one form migrating like the protein purified from the envelope, another (the most abundant form) migrating faster, and a third migrating between those two (Fig 9, panel C, Coomassie blue stained gel (CB), lanes 5–8). After CuAAC, we found that only the upper band gave a signal, whether isolated from the envelope or the supernatant (Fig 9, panel C, Streptavidin-HRP detection). This result indicates that mycoloylated PorC is mostly located in the envelope whereas a large quantity of non-mycoloylated protein (non-labeled, lower migrating form) is released in the supernatant. However, to ensure that the faster migrating form of PorC_his_ isolated from the supernatant is indeed representative of a distinct mycoloylation status instead of proteolytic degradation, we treated the protein isolated from the envelope or supernatant with NaOH. Alkaline treatment is indeed known to cleave ester bonds and remove mycolates form *O*-mycoloylated proteins [6]. Here, alkaline treatment resulted in the complete conversion of PorC_his_ isolated from the envelope (upper band) into a protein migrating at the same position in the gel as the protein isolated from the culture supernatant (Fig 9, panel D). In addition, the two minor upper bands observed in PorC_his_ purified from the supernatant were also converted to the non-modified form upon treatment with NaOH. This experiment hence confirms that PorC_his_ is mycoloylated in the envelope whereas the major form present in the culture supernatant is not. Together with the results obtained with PorB, which is only labelled when purified from the envelope, these data strongly suggest that mycoloylation has a role in PorC and PorB envelope association.

## Discussion

### Generality of the mycoloylation process

Mycoloylation has been seldom studied so far and it is not known how widespread this modification is in *Corynebacteriales*. Here, we found that, in addition to the two porins PorA and PorH, two other pore-forming proteins, PorB and PorC, are also mycoloylated. These results are the first evidence for a post-translational modification of these two proteins and suggest that in *C*. *glutamicum*, mycoloylation is not restricted to PorH, PorA and ProtX but could be a more general process. It remains to be determined whether additional proteins are mycoloylated in *C*. *glutamicum* or other *Corynebacteriales*. Interestingly, post-translationally modified porins have also been described in *Mycobacterium*. An oligomeric porin has recently been identified in *Mycobacterium marinum* (MMAR_0617) that lacks a signal-sequence and which activity and membrane association is dependent on a threonine-rich C-terminal domain. This domain appears to be extensively modified, resulting in the doubling of the protein apparent molecular weight on SDS-Page gel. Although the nature of the modification was not definitively identified, its alkaline sensitivity suggested an *O*-glycosylation or/and *O*-mycoloylation [55]. However, the conservation of the mycoloylation pathway in other *Corynebacteriales* such as Mycobacteria, is not demonstrated. MytC was shown to differ from other mycoloyltranferases by the presence of a loop that would define the specificity of this enzyme for peptide rather than sugars [30]. Orthologues of *mytC* have been found in the genera *Nocardia*, *Gordonia* and *Rhodococus* but these enzymes were not all active in *C*. *glutamicum* [30]. In contrast, none of the *Mycobacterium* identified mycoloyltransferases have been showed to possess the MytC-characteristic loop, suggesting that proteins mycoloylation may not occur in this genus. However, this remains to be tested. For this purpose, metabolic labeling represents a very useful tool, to either test specific candidates or to perform global identification of a given bacterium mycoloylome.

### Role of protein mycoloylation

Protein lipidation frequently allows anchoring of otherwise soluble proteins to membranes. Here, we found that in the case of PorB and PorC, mycoloylation indeed appears to favor association to the envelope. However, in the case of PorA and PorH, mycoloylation appears not to fullfill such a function as in the *mytC*::*Km* mutant, PorA is still associated with the envelope and not detected in the culture supernatant (not shown). This could not be directly tested for PorH because the protein is poorly expressed in this strain. However, similar to the non-mycoloylated mutant PorA_his_-S15V, the PorH_his_-S56A mutant is not released from the wild-type strain envelope (not shown), which indicates that PorA and PorH can remain associated with the mycomembrane independently of their mycoloylation status. The Kyte and Doolittle hydrophilicity plots of these proteins indeed indicate a relatively high proportion of hydrophobic residues, that could support a lipidation-independent mycomembrane localization. Similarly, PorB and PorC being porins, it is expected that these proteins would be integral mycomembrane proteins rather than soluble proteins anchored to the membrane by the covalent attachment of a lipid. However, the mechanism by which these proteins would be targeted and inserted into the mycomembrane is not known. Interestingly, several lipidated outer membrane proteins, including α-helical, multimeric pore-forming complexes have been shown to be assembled in the outer membrane of Gram-negative bacteria by the lipoprotein sorting pathway (Lol) rather than by the more general β-barrel assembly machinery (Bam system) [56]. As no homologues of the Bam or Lol systems have been identified in *Corynebacteriales*, it is possible that in *C*. *glutamicum*, a system exists that depends on mycoloylation for targeting, insertion and/or proper assembly of certain proteins in the mycomembrane. Potentially, PorB and PorC could be substrates of such a system.

Still, protein lipidation does not fulfill only a role in membrane targeting, insertion or anchoring. In eukaryotes, some integral membrane proteins possessing multiple transmembrane domains are *S*-palmitoylated on cytosolic loops or tails, which are thus brought back up to the membrane when acylated. This in turn affects these loops/tails conformation and can modulate protein conformation, protein function and/or interaction with other proteins [57–60]. Interestingly, ion channels appear to be major targets of *S*-palmitoylation in eukaryotic cells and the modification controls both surface expression and intrinsic activity of these proteins [61]. As PorHA *in vitro* porin activity is dependent on PorA mycoloylation, it is hence possible that PorA modification is important either for association with PorH, or for PorA to adopt an active conformation in the mycomembrane. The function of PorH mycoloylation is more puzzling, as it is apparently not necessary for PorHA porin activity *in vitro*, nor for association to the envelope.

In contrast with PorC, a pore-forming activity has been demonstrated for PorB [26]. Future work will thus focus on determining whether PorC also displays porin activity *in vitro* and whether PorB (and eventually PorC) activity is dependent on mycoloylation or/and association between PorB and PorC. Interestingly, serine-rich regions, located at the N and C-terminal ends of PorB, were found to extend out from PorB core structure without a defined fold. The clear sequence conservation of these extensions between species led Ziegler *et al*., (2008) [27] to propose that they could play an important role in oligomerization. If these regions include the site of mycoloylation, it is tempting to speculate that mycoloylation could hence be involved in the oligomerization of these porins.

Finally, we found that in our growth conditions (culture in BHI rich medium), PorH_his_ could not be over-expressed and purified from the Δ*mytC*::*Km*, Δ*pks13*::*Km* or CgΔ*porHporA* strains, whereas both PorH_his_ and PorH_his_-S56A could be readily expressed and purified from the wild-type strain. Endogeneous PorH could also only be detected in the wild-type strain by western-blot analysis (not shown). In contrast, a peak corresponding to non-modified PorH was observed by MALDI-TOF analysis of the Δ*mytC*::*Km* and Δ*pks13*::*Km* organic solvants extracts. This peak, however was repeatedly smaller in the Δ*mytC*::*Km* strain than in the wild-type strain [6,30] suggesting that PorH level is indeed reduced in these strains.

### Mycoloylation pathway

Together with the exact role of protein mycoloylation, the process of mycoloylation itself needs to be further studied. Whether the enzyme responsible for this modification (most probably MytC) recognizes an amino acid sequence or a structural motif in the protein substrate is not known. Here, we identified ProtX mycoloylation site as being the penultimate residue of the protein, Ser37. Hence, the modification of this protein also occurs on a serine residue as for PorH and PorA. Whether some mycoloylated proteins are modified on threonine or tyrosine residues is unknown.

The 3 sites of mycoloylation determined so far do not share any surrounding sequences that would constitute an obvious mycoloylation consensus motif. The generation of a larger dataset of experimentally determined mycoloylation sites is needed to statistically determine whether certain residues have increased frequency of occurrence near the mycoloylated serines. These results would then be used to design a method to predict the chance for a position to be mycoloylated. Such an approach, which requires the identification of large number of mycoloylated proteins, has previously been applied to *O*-glycosylation or *S*-palmitoylation, for which no specific sequence motifs are defined [62,63].

Nine and 10 serine residues are present in the mature forms of PorB and PorC respectively, and most of these residues are clustered in the conserved serine-rich regions at the N and C-terminus of the proteins [26,27]. Interestingly, the four C-terminal residues of PorB (_123_NFSS_126_) are identical to the PorH C-terminal sequence that includes its mycoloylation site (_54_NFSS_57_). The penultimate serine residue of PorB (Ser125) is hence a good candidate to test as a potential mycoloylation site.

Another remaining question is when during protein biogenesis and where in the envelope, mycoloylation takes place. MytC, like all its targets identified to date, is associated with the mycomembrane [47,54]. The probable mycolate donor of the reaction (TMM) being also mainly located in the mycomembrane, we can imagine that the transfer occurs in this environement. An alternative hypothesis is that mycoloylation occurs immediatly after export through the cytoplasmic membrane and serves as a signal for the mycoloylated proteins to reach the mycomembrane.

### Metabolic labeling of mycoloylated proteins

Metabolic labeling is a powerful approach which, when coupled to click-chemistry reactions, allows the rapid visualization or isolation of molecules that cannot be genetically tagged. This approach has already proved useful in the characterization of various phenomena occurring at the bacterial cell surface, as well as in the proteomic identification of post-translationally modified proteins [34–36,64]. Here, we show that an alkynyl-fatty acid analogue (17-ODYA) can be taken up by *Corynebacterium glutamicum* and used as a substrate to label mycoloylated proteins. The absence of labeling in a strain that cannot produce mycolic acids supports the assumption that 17-ODYA is transported to the cytoplasm and used by Pks13 to produce modified mycolic acids, which are then transported back to the envelope and transferred onto target proteins in a MytC-dependent process (Fig 1). Here, we used this detection method to test potential candidates of interest (PorB and PorC). However, another advantage of metabolic labeling compared to other detection methods, is that it allows global proteome profiling. Specifically, the incorporated group (here alkynyl) can be linked to an affinity handle for enrichment, followed by identification by mass spectrometry. The labeling of PorA, PorH and ProtX observed here is a proof of concept suggesting that the “mycoloylome” of *C*. *glutamicum* could be identified by this approach. However, 17-ODYA, like any fatty acid analogues, will most probably also be incorporated in phospholipids and thus transferred to “classical lipoproteins” and eventually to any other proteins modified with fatty acids (other “non canonical” lipidated proteins). Metabolic labeling with 17-ODYA will hence permit the enrichment of all fatty-acid-modified proteins from *C*.*glutamicum*. To circumvent this difficulty and in order to isolate mycoloylated proteins from lipoproteins, two strategies are available. First, mycoloylated proteins could be labeled and enriched from a strain that do not acylate classical lipoproteins. Indeed, the biogenesis pathway for classical lipoprotein is well described and the enzymes responsible for lipoproteins modification are known. In particular, absence of the first enzyme of the pathway, Lgt (prolipoprotein diacylglyceryl transferase), results in the total absence of lipoproteins acylation [3]. In *C*. *glutamicum*, deletion of *lgt* does not affect viability and has no effect on PorA mycoloylation (not shown). The *Cg*Δ*lgt* mutant could hence be used for the isolation and identification of mycoloylated proteins together with potential other “non canonical” lipidated proteins, if such proteins exist in *C*. *glutamicum*. Another possibility would be to use, instead of a fatty acid analogue, an alkynyl analogue of trehalose monomycolate (TMM), TMM being the probable substrate used by MytC for mycoloylation. Incidentally, during the course of this study, a TMM mimic with a shortened akynyl lipid chain has been described and showed to access *Mycobacterium smegmatis* and *C*. *glutamicum* periplasm where it is used by mycoloyltransferases (Ag85) to acylate arabinogalactan and TMM [65]. While our manuscript was in revision, a whole-cell proteomic approach was published where the authors used this alkynyl-TMM probe to identify mycololyted proteins in *C*. *glutamicum* [66]. Although most of the putative mycoloylated proteins identified in this study need further confirmation, it is interesting to note that PorB was part of the identified candidates and suggested to be modified on multiple sites. In contrast, PorC was not retrieved in this screen, demonstrating the necessity to use complementary approaches to obtain exhaustive results.
